# Clean fleets, different streets: evaluating the effect of New York City’s clean bus program on changes to estimated ambient air pollution

**DOI:** 10.1038/s41370-022-00454-5

**Published:** 2022-07-20

**Authors:** Gina S. Lovasi, Christian A. Treat, Dustin Fry, Isha Shah, Jane E. Clougherty, Alique Berberian, Frederica P. Perera, Marianthi-Anna Kioumourtzoglou

**Affiliations:** 1 Department of Epidemiology and Biostatistics, Urban Health Collaborative, Dornsife School of Public Health, Drexel University, Philadelphia, PA USA; 2 Columbia University Vagelos College of Physicians and Surgeons, New York, NY USA; 3 Columbia Center for Children’s Environmental Health, Mailman School of Public Health, Columbia University, New York City, NY USA; 4 Department of Environmental and Occupational Health, Dornsife School of Public Health, Drexel University, Philadelphia, PA USA; 5 Department of Environmental Health Sciences, Mailman School of Public Health, Columbia University, New York City, NY USA

**Keywords:** Air Pollution, Particulate Matter, Epidemiology, Criteria Pollutants, Environmental Justice, Geospatial Analyses

## Abstract

**Background:**

Motor vehicles, including public transit buses, are a major source of air pollution in New York City (NYC) and worldwide. To address this problem, governments and transit agencies have implemented policies to introduce cleaner vehicles into transit fleets. Beginning in 2000, the Metropolitan Transit Agency began deploying compressed natural gas, hybrid electric, and low-sulfur diesel buses to reduce urban air pollution.

**Objective:**

We hypothesized that bus fleet changes incorporating cleaner vehicles would have detectable effects on air pollution concentrations between 2009 and 2014, as measured by the New York City Community Air Survey (NYCCAS).

**Methods:**

Depot- and route-specific information allowed identification of areas with larger or smaller changes in the proportion of distance traveled by clean buses. Data were assembled for 9670 300 m × 300 m grid cell areas with annual concentration estimates for nitrogen oxide (NO), nitrogen dioxide (NO_2_), and black carbon (BC) from NYCCAS. Spatial error models adjusted for truck route presence and total traffic volume.

**Results:**

While concentrations of all three pollutants declined between 2009 and 2014 even in the 39.7% of cells without bus service, the decline in concentrations of NO and NO_2_ was greater in areas with more bus service and with higher proportional shifts toward clean buses. Conversely, the decline in BC concentration was slower in areas with more bus service and higher proportional clean bus shifts.

**Significance:**

These results provide evidence that the NYC clean bus program impacted concentrations of air pollution, particularly in reductions of NO_2_. Further work can investigate the potential impact of these changes on health outcomes in NYC residents.

**Impact Statement:**

Urban air pollution from diesel-burning buses is an important health exposure. The New York Metropolitan Transit Agency has worked to deploy cleaner buses into their fleet, but the impact of this policy has not been evaluated. Successful reductions in air pollution are critical for public health.

## Introduction

Despite federal and municipal attempts to curb emissions, urban air pollution from traffic remains a top public health concern [1]. While use of public transit can reduce emissions from personal automobiles, emissions from diesel buses themselves can be a source of air pollution, including nitrogen oxides (NO_x_) and black carbon (BC) [2, 3]. Changes to the types of fuels used and other emission-related technology have the potential to reduce the air quality impacts of public transit vehicles and reduce exposure disparities. In the US, federal regulation since 1988 has stimulated local bus fleets to meet incrementally more stringent emissions standards, and governments globally are also taking a leading role in addressing the challenge of bus-related urban air pollution [4, 5].

Quantifying the impact of local shifts toward lower-emission vehicles on the urban environment can provide impetus to expand or refine such efforts by putting the bus fleet-attributable changes in context alongside changes in other spatially-patterned emission sources. Bus fleet modernization and subsequently improved urban air quality could help reduce adverse health outcomes that have been associated with traffic-related air pollution, such as asthma hospitalization, preterm birth, low birthweight, and cardiopulmonary mortality [6–8]. Previous studies modeling benefits of shifting toward a cleaner bus fleet for a given urban area have estimated emissions reductions, avoided healthcare costs, and reduced mortality [9, 10]. However, emissions, and thus the potential benefits from reducing emissions from a given source, vary within a given city, and these variations have not been adequately assessed.

Within New York City (NYC), locations with bus routes or higher total traffic volume have been associated with higher annual nitrogen dioxide (NO_2_) levels, while other pollutants such as sulfur dioxide (SO_2_) appeared to be more sensitive to spatial variation in non-vehicle sources such as heating oil in residential buildings [11]. Isolating the contribution of local bus fleet changes in the context of a complex urban environment is challenging but can benefit from characterization of the timeline of fleet change, as well as the spatial distribution of bus routes and bus service intensity. Specifically, bus fleet changes are expected primarily to affect concentrations of traffic-related pollutants such as NO_x_. Further, because bus fleet changes are not uniformly distributed, the effect is expected to be greatest for those areas served by depots that have had a relatively greater shift toward service by clean vehicles.

In this paper, we sought to understand the effects of the Clean Fuel Bus Program from 2009 to 2014 on the spatial distribution of emissions and air pollution concentration changes. In brief, the Clean Fuel Bus Program sought to reduce emissions from the bus fleet by purchasing new, lower-emissions buses including compressed natural gas (CNG), hybrid-electric, and ultra-low-sulfur diesel (USLD) buses. In addition, existing buses were retrofitted with diesel particulate filters (see Box 1). Any measurable benefits to air quality from these changes could either be associated with overall bus traffic across the city, or—if the implementation of fleet changes were not evenly spatially distributed—associated with area-specific fleet shifts.

Depot- and route-specific information including fuel type and bus model year was assembled along with air pollutant concentration data from the New York City Community Air Survey (NYCCAS) for 300 m × 300 m grid-cell areas. We hypothesized that areas with more bus traffic and areas with the greatest shift toward CNG, hybrid-electric, ultra-low-sulfur diesel, as well as post-2007 vintage buses would experience relatively greater declines in nitrogen oxide (NO), nitrogen dioxide (NO_2_), and black carbon (BC) levels. Support for this hypothesis could provide a foundation for future work linking bus fleet change to longitudinal population health outcomes.

Box 1 Overview of federal and municipal policy context for the NYC bus fleet changes Federal efforts to reduce emissions from transit buses in the United States were initiated under the Clean Air Act of 1990 [26]. This law set emission standards for all new bus purchases, beginning with those built in 1998 onwards. Further, select cities, including NYC, were mandated to purchase clean fuel buses.

As clean fuel bus programs grew and expanded across cities, the vision of what constituted a clean bus varied [13]. Often, local transportation agencies adopted “replace and remediate” strategies, an incremental process of replacing older buses with newer models and retrofitting existing buses with emission reduction technologies. Other strategies considered by some transportation agencies aimed to shift away from using buses that operate exclusively on diesel. Initially, fleet conversion to compressed natural gas (CNG) and hybrid-electric buses were favored as cleaner alternatives to diesel [27]. Later, when federal mandates lowered the maximal sulfur content in diesel fuel, some local transit agencies reverted to conventional diesel buses while shifting to reliance on fuel with a lower-sulfur content, referred to as ultra-low sulfur diesel (ULSD) buses [28].

There is considerable intercity variation in United States Clean Bus Programs. Los Angeles has the second-largest bus system in the United States and exclusively operates with CNG buses [29]. NYC has the largest bus system in the United States and started updating their fleet to reduce emissions beginning in 2000 using a combination of methods [18]. From that point onward, new CNG, hybrid-electric, and ULSD buses were purchased to replace aging buses (buses typically remain in use for about 12 years), and existing buses were retrofitted with diesel particulate filters and repowered with newer engines [12]. In 2005, all MTA buses were powered by newer diesel engines [12], and by mid-2006, 3,508 diesel buses had been installed with diesel particulate filters [30], representing 89% of the diesel-powered fleet in that year. These interventions resulted in substantial decreases in estimated emissions between 1995 and 2006 [30].

## Methods

### Setting and study overview

For our analyses, we assembled depot and route data to estimate characteristics of bus service and bus fleet composition in 2009 and 2014 throughout the five boroughs of NYC. The geographic units used were 9670 grid cells (approximately 300 m × 300 m) drawn over land to align with the availability of pollutant concentration estimates from NYCCAS [10, 12].

### Outcome variables from the New York City Community Air Survey

Predicted NO, NO_2_, and BC concentrations at the sub-city level were based on raster surfaces from the NYCCAS of estimated annual-average pollutant concentrations, produced using land-use regression that incorporated extensive measures of traffic and truck intensity, industrial facilities, built environment characteristics, and population metrics as described previously [13]. The outcome of interest was the difference in estimates from 2009 to 2014 (i.e., NO_2014_–NO_2009_) for each grid cell. NO and NO_2_ are likely to be sensitive to the localized variation in bus fleet emissions across this period, based on sharp concentration decay curves alongside roadways [14–16]. BC is also considered here for a more complete understanding of the impact of bus fleet changes on pollutant concentrations, as the diesel engines in buses tend to produce more particulate matter air pollution than do light-duty gasoline vehicles such as personal automobiles [17]. Further, early testing of NYC’s hybrid buses found that hybrid bus models may produce more hydrocarbon pollution than do conventional diesel buses under some conditions [18], so results may differ for BC when compared to NO_X_.

### Assessment of bus service characteristics

In NYC, each route is served by a designated bus depot, and newly purchased buses are assigned to a bus depot based on factors including fuel types supported by the depot and service demands. While the depots changed somewhat over our study period, there were 27 operational depots in both 2009 and 2014. The median depot served 12 routes in 2009 and 11 in 2014, with some routes being assigned to more than one depot.

Depot-level information on yearly bus fleet assignment rosters were obtained through a Freedom of Information Act request. Information obtained included the count of buses by fuel type (hybrid electric, CNG, and ULSD) and model year. Information on bus fleet composition and routes served at the depot level was used to characterize the mix of buses providing service to a given grid cell area, assuming that all routes served by a given depot had the same proportion of service by each bus type. When routes were served by more than one depot, bus types were evenly apportioned from each depot serving that route.

We considered two criteria of a “clean” bus. Our primary definition included hybrid-electric, CNG, and ULSD buses, in addition to any buses with a model year 2007 or later, which are subject to stricter emissions standards than pre-2007 buses (“broad definition”). To understand the degree to which our results were sensitive to the inclusion of ULSD and recent vintage buses, we created a secondary, narrower definition to include hybrid-electric and CNG buses only (the “narrow definition”). This allows us to assess the effect of NYC-specific policies separately from the federal level, as the NYC policies affected the procurement of CNG and hybrid-electric buses while federal legislation is responsible for emissions changes in later-vintage buses. The broad category of buses includes both sources of changes while the narrow category is specific to changes at the local level.

Bus route maps were available in geographic information systems (GIS) formats for years 2009 [19] and 2012 [20], and the 2012 route map was used for 2014 (year-on-year changes to the bus maps are minimal). The average annual number of buses serving each route was estimated from their weekday and weekend frequency as determined from the MTA’s public bus service guides. Annual vehicle meters traveled (VMT) for a given route was calculated by multiplying the GIS-derived bus route length that fell within each grid cell by the average annual number of buses on that route.

We estimated the proportion of bus vehicle meters traveled (VMT) served by clean buses in both 2009 and 2014 and calculated the difference in the proportion of clean buses between the same years:

$$\Delta Prop.Clean\,Bus\,VMT = \ Clean\,Bus\,VMT_{2014}/Total\,Bus\,VMT_{2014}\\ - \,Clean\,Bus\,VMT_{2009}/Total\,Bus\,VMT_{2009}$$

### Potentially confounding traffic characteristics

To assess and account for potential confounding by other traffic sources, publicly-available data sources previously noted to predict local pollutant concentrations were obtained [9, 11]. Total traffic volume, consisting of all on-road motor vehicles, was obtained for each grid cell using data from the New York Metropolitan Transportation Council [21]. The presence of truck routes in each grid cell was assessed using the NYC Truck Route map [22].

### Statistical analysis

Descriptive statistics and spatial regression at the level of the 300 m grid cell were used to explore area-level associations of bus service and traffic characteristics with the difference in NYCCAS estimates from 2009 to 2014 for NO, NO_2_, or BC as the dependent variable. Spatial error models were used to account for non-independence of grid cells. The change in proportion of clean fleet shift was dichotomized at the median for descriptive purposes and treated continuously in regression models. Adjusted models included total road traffic volume and the presence of a truck route in the grid cell, with (Model 3) or without (Model 2) controlling for total bus traffic volume at 2009 levels. Both total road traffic and bus traffic were log transformed in all models due to a strong right skew in both variables.

All geographic analyses were performed in ArcGIS, and R version 4.0.0 was used for all data processing and statistical models. Spatial error models were computed using the spatialreg package [23].

## Results

**Fig. 1:**
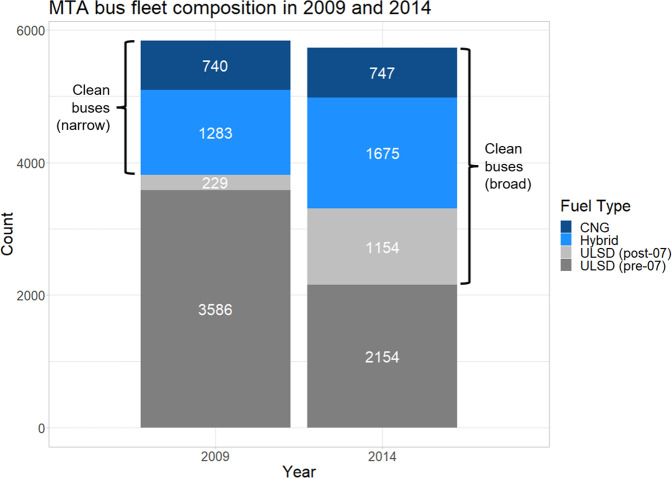
MTA bus fleet composition in 2009 and 2014. The proportion of buses classified as “clean” increased between 2009 and 2014, whether considering the narrow or broad clean bus criteria.

From 2009 to 2014, the total fleet size was stable (decreasing slightly from 5838 to 5730 buses, as shown in Fig. 1). During this period, the proportions of CNG buses remained similar in both years and ULSD buses decreased from 65.3% to 57.7%. The greatest changes toward clean buses were the increase in hybrid-electric buses from 22.0% to 29.2% and the increase in the proportion of post-2007 model year buses from 16.9 % of the total fleet to 48.7%.

**Fig. 2:**
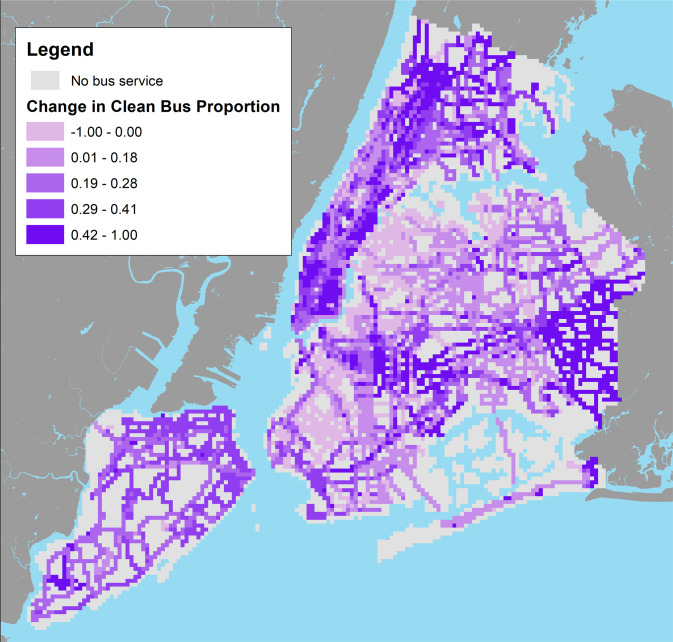
Proportional changes in the distance traveled by clean buses were not evenly spatially distributed across the city. Cell-specific changes in the proportion of VMT by clean-fuel buses (broad definition). Positive values represent a proportional increase in clean bus proportion over the time period. Cells with no bus traffic in either year correspondingly had no change in bus type and are greyed out on this map.

Of the 9670 grid cell areas included, 3839 (39.7%) did not have bus service in either 2009 or 2014. Areas with bus service were more likely to have truck routes present and higher total traffic volume (Table 1, Fig. 2). Across all grid cells with bus service in both 2009 and 2014, the median increase in the proportion of VMT by clean buses was 23% (6% using the narrow definition of clean buses). On average, the changes in NO, NO_2_, and BC concentrations from 2009 to 2014 were negative in each of our exposure groups, indicating that traffic-related pollutant concentrations were decreasing for this time period as previously documented. Supplementary Table 2 displays descriptive information for all cells considering the narrow clean fleet definition rather than the broad fleet definition.

**Table 1:** Decreases in air pollutant concentrations are of similar magnitude in grid cells regardless of their clean fleet change category.

	No bus service	At or below median clean shift	Above median clean shift
	*N* = 3839	*N* = 2931	*N* = 2900
	Mean (SD)	Mean (SD)	Mean (SD)
**Bus Traffic**			
Δ Clean VMT 2009-2014		4.1% (18.49%)	37.44% (11.4%)
Clean VMT, 2009 (km)		1.5780e + 04 (2.0562e + 04)	7.9430e + 03 (1.3782e + 04)
Clean VMT, 2014 (km)		2.1677e + 04 (2.6638e + 04)	2.1306e + 04 (2.4468e + 04)
Total VMT, 2009 (km)		3.0730e + 04 (4.3848e + 04)	3.0540e + 04 (4.2462e + 04)
Total VMT, 2014 (km)		3.4599e + 04 (4.2917e + 04)	3.4692e + 04 (3.9895e + 04)
Number of bus stops	0.0049, (0.0806)	2.566 (2.4075)	2.859 (2.4077)
**Other traffic sources**			
Truck route present	9.74%	60.32%	70.34%
Total traffic VMT (km)	2.6932e + 06, (7.5584e + 06)	1.0553e + 07 (1.3569e + 07)	7.8205e + 06 (1.1067e + 07)
**Pollutant concentrations**			
Δ NO 2009–2014 (ppb)	−3.0775 (1.5459)	−3.6815 (1.8919)	−4.1643 (2.5428)
Δ NO_2_ 2009–2014 (ppb)	−2.9695 (1.3797)	−4.1414 (1.5173)	−4.0518 (1.7431)
Δ BC 2009–2014 (ppb)	−0.177 (0.0298)	−0.184 (0.0233)	−0.1774 (0.0322)

Values representing a proportion are expressed as a percentage. Values representing a magnitude are expressed as mean (standard deviation) across all grid cells in each respective category. Clean fleet changes tend to co-occur with higher total traffic volume and presence of a truck route.

Modeling suggests an impact of the bus fleet changes on concentrations of all assessed pollutants, but the direction of association was not consistent (Table 2). Declines in the concentration of NO and NO_2_ were greatest in grid cells with higher levels of bus traffic (Model 3), although the magnitude of effect was very small. Negative associations between cell-specific clean shifts and pollutant concentration changes were present in models controlling for truck route presence and total traffic volume (Model 2), but while this association persisted with the addition of total bus traffic in the NO_2_ model, it did not persist in the NO model. The direction of association was reversed in BC models. BC concentrations saw *less* improvement in grid cells with proportionally greater shifts toward clean bus technology (Model 2) and in cells with more overall bus traffic (Model 3). Results from unadjusted models are reported in Supplementary Table 1.

**Table 2:** Pollutant concentration changes are associated with total bus traffic and proportional clean shifts.

	Δ NO		Δ NO_2_		Δ BC	
	Model 2	Model 3	Model 2	Model 3	Model 2	Model 3
**Δ prop clean buses**	−0.04947^a^ (−0.0911, -0.00787)	−0.0176 (-0.0617, 0.02660)	-0.01084^a^ (−0.01587, -0.00580)	−0.00758^a^ (−0.01293, −2.2e−03)	7.1e−05^a^ (2.4e−05, 1.2e−04)	4.7e−05 (−3.1e−06, 9.8e−05)
**Total traffic 2010**^b^	−0.00069 (−0.0016, 0.00025)	−0.0003 (−0.0013, 0.00066)	−0.00014^a^ (−0.00026, −0.00003)	−0.00010 (−0.00022, 1.2e−05)	−1.8e−07 (−1.3e−06, 9.0e−07)	−4.8e−07 (−1.6e−06, 6.2e−07)
**Truck route**	−0.03518^a^ (−0.0530, −0.01734)	−0.0203^a^ (−0.0394, −0.00114)	0.00163 (−0.00053, 0.00379)	0.00315^a^ (0.00083, 5.5e−03)	3.3e−05^a^ (1.3e−05, 5.4e−05)	2.2e−05^a^ (2.1e−07, 4.4e−05)
**Bus VMT 2009**^b^		−0.0020^a^ (−0.0030, −0.00109)		−0.00021^a^ (−0.00032, −9.3e−05)		1.5e−06^a^ (4.4e−07, 2.6e−06)

Beta coefficients and 95% confidence intervals presented. Negative coefficients indicate that pollutant concentrations declined more quickly with increasing levels of the independent variable, while positive coefficients indicate that increasing levels of the independent variable were associated with slower declines in pollutant concentrations.

^a^Association significant at *p* < 0.05.

^b^Log-transformed variable.

Among the pollutants considered, NO_2_ was the most consistently associated with bus-fleet-related improvements. Similar associations were found for NO_2_ models when considering a narrowly-defined clean fleet criterion that included only hybrid and CNG buses, but results diverged for NO and BC models (Supplementary Table 3). Supplementary Table 4 displays results for unadjusted models using the narrow fleet definition.

## Discussion

In this area-level analysis of declining estimated annual pollutant concentrations from 2009 to 2014 in NYC, a shift toward clean bus service was associated with measurably greater improvements in local NO and NO_2_ concentrations. Results for NO_2_ are robust to different definitions for “clean bus service” that reflect NYC’s combined strategy for fleet modernization, with significant associations being found in models using both broad and narrow clean bus definitions. However, shifts toward clean bus service were associated with slower declines in BC concentration. An explanation for this finding may be found in previous work suggesting that hybrid diesel buses can produce more particulate matter than their conventional counterparts due to their smaller engine size [24]. Alternately, it may be an artifact of our fine-scale study design: because nitrogen oxides have a sharper decay curve from the roadway when compared to black carbon, our models may have been less resilient against residual confounding in the black carbon models.

The results of this analysis should be interpreted with attention to several limitations. Due to the focus on a change in annual concentration estimates over a 5-year period, temporal variation in both the bus service (including service changes, reroutes, or bus reassignment to other depots within a given year) and pollutant concentrations were not completely captured. This analysis also did not include all transit vehicles on NYC streets, such as paratransit services offered through the same transit agency or bus fleets maintained by private companies (e.g., those providing service between cities). In addition, our measure for traffic volume is only for a single year, and so may not adequately control for variation in traffic concentrations in space over time. Finally, our study captures variation only in air pollution, without considering variation in population. Reductions in traffic emissions may have a greater magnitude of effect on overall population health in areas of the city where more people reside, in areas of concentrated disadvantage, or on streets where a high volume of commuters are frequently travelling.

It is also important to consider that NO_x_ tailpipe emissions can evolve into health-relevant secondary particulate matter concentrations [25], but this particulate matter would be more dispersed, with less spatial association with bus traffic than tailpipe NO_x_ emissions. Such changes in secondary pollutant concentrations as a result of bus fleet changes would therefore not be detected by these methods. However, they may represent a plausible mediational pathway between bus fleet changes and health outcomes.

In conclusion, although the effect of different bus technologies and bus service on health outcomes remains unassessed, the observed associations with intra-urban air pollution reductions suggest that health benefits of bus fleet shifts within NYC are plausible, and that NO_x_ concentrations (and NO_2_ in particular) are more likely than BC tailpipe emissions to mediate such benefits. Although transportation agencies must balance considerations including cost, ridership demand, and fleet availability when planning service and route changes, when viewed alongside prior work [9, 10] our findings suggest that air quality stands to benefit from introducing cleaner buses into the existing fleet.

## Supplementary information

Supplementary information: table captions

Supplementary tables

## Data Availability

Variables related to the bus fleet and bus traffic were generated by the authors based on publicly-available data and FOIL requests, and are available upon request of the authors. The New York City Community Air Survey pollutant data used in this analysis are publicly available from NYC OpenData. New York Metropolitan Transit Council traffic estimates are proprietary and held at the discretion of NYMTC.
