# Supplementary material for: Clean fleets, different streets: evaluating the effect of New York City’s clean bus program on changes to estimated ambient air pollution
Source: J Expo Sci Environ Epidemiol. 2022 Jul 20;33(3):332–8. doi: 10.1038/s41370-022-00454-5 (PMC10234802; doi:10.1038/s41370-022-00454-5)
Supplement: Supplementary file 1 — Supplementary information: table captions [file 41370_2022_454_MOESM1_ESM.docx]

**Supplementary Table Captions**

**Table 1**: In uncontrolled models, changes in pollutant concentrations are associated with clean bus fleet shifts under the broad definition.

*****Association significant at p<0.05

**Table 2**: Considering the narrow fleet definition, there is a trend for greater decreases in NO and NO_2_ concentrations in cells with greater proportional clean shifts. Greater proportional clean shifts do not appear to be associated with either the presence of a truck route or total traffic.

Values representing a proportion are expressed as a percentage. Values representing a magnitude are expressed as mean (standard deviation) across all grid cells in each respective category.

**Table 3**: NO_2_ concentration changes are associated with total bus traffic and proportional clean shifts when considering the narrow clean fleet definition.

Beta coefficients and 95% confidence intervals presented. Negative coefficients indicate that pollutant concentrations declined more quickly with increasing levels of the independent variable, while positive coefficients indicate that increasing levels of the independent variable were associated with slower declines in pollutant concentrations.

*****Association significant at p<0.05

**^†^**Log-transformed variable

**Table 4**: In uncontrolled models, changes in NO_2_ concentrations are associated with clean bus fleet shifts under the narrow definition.

*****Association significant at p<0.05
